# Effects of focused electron beam irradiation parameters on direct nanostructure formation on Ag surfaces

**DOI:** 10.3762/bjnano.13.87

**Published:** 2022-09-22

**Authors:** Jānis Sniķeris, Vjačeslavs Gerbreders, Andrejs Bulanovs, Ēriks Sļedevskis

**Affiliations:** 1 Daugavpils University, Institute of Life Sciences and Technologies, Parādes Str. 1, Daugavpils, LV-5401, Latvia

**Keywords:** atomic force microscopy, electron beam, lithography, nanostructure, silver, sputtering, surface

## Abstract

Metallic nanostructures are applied in many fields, including photonics and plasmonics, due to their ability to absorb or emit light at frequencies which depend on their size and shape. It was recently shown that irradiation by a focused electron beam can promote the growth of nanostructures on metal surfaces and the height of these structures depends on the duration of the irradiation and the material of the surface. However, the effects on growth dynamics of numerous irradiation parameters, such as beam current or angle of incidence, have not yet been studied in detail. We explore the effects of focusing, angle of incidence, and current of the electron beam on the size and shape of the resulting structures on an Ag surface. In addition, we investigate how the nitrogen plasma cleaning procedure of a vacuum chamber can affect the growth of these structures. A beam current of around 40 pA resulted in the fastest structure growth rate. By increasing the beam diameter and angle of incidence the growth rate decreased; however, by raising the beam focus up to 5–6 μm above the surface the growth rate increased. Vacuum chamber cleaning reduced structure growth rate for a few hours. These findings can help to better control and optimise the growth of nanostructures on metal surfaces undergoing irradiation by a focused electron beam.

## Introduction

Metallic nanostructures have various uses, including in nano-electro-mechanical systems [1], plasmonic biosensors [2], and nanophotonics [3]. They can also serve as catalysts for controlled chemical vapour deposition [4]. While gold is the most widely used material for fabrication of plasmonic nanostructures, silver can offer a less expensive alternative [5–7].

Electron beam (EB) lithography is a popular method for the nanopatterning of metal surfaces, but it is a complicated and expensive multistep process [8]. Electron beam induced deposition (EBID) is a direct-write lithography technique, which is capable of creating 2D and free-standing 3D nanostructures by using electron irradiation to dissociate volatile precursor molecules, whose products of dissociation are deposited on the irradiated area [9–11]. Normally, precursor molecules are intentionally delivered to the irradiated area by a gas flow. However, residual gases in the vacuum chamber can also be used as a precursor for EBID. Nanostructures produced from residual hydrocarbons by electron irradiation in scanning electron microscopy (SEM) vacuum chambers have been reported in several studies [12–15].

Hydrocarbon contamination from samples and vacuum pump oils is known to be ever present in vacuum chambers of electron microscopes [16–18]. The energy required to break atomic bonds in hydrocarbons can be quite low (less than 5 eV) [19]. Therefore, even low-energy secondary electrons (SE), which are released from the surface around the point of impact of the EB, are capable of dissociating hydrocarbon molecules. Secondary electrons are usually emitted from an area much larger than the size of EB. The size of this area and the amount and energy of emitted SE depend on several factors, including the energy, current, and angle of incidence of the EB, as well as the material and thickness of the target [20–21]. The formation of carbon layers is a common, but often undesirable occurrence on surfaces irradiated with EB [22–23]. In high-vacuum conditions, hydrocarbons tend to get adsorbed onto the surfaces of vacuum chamber or samples, where they can move via thermally activated diffusion. Cooling the surface can immobilize any adsorbed molecules and prevent a buildup of carbon in the irradiated area. Baking can be used to desorb light molecules with a high vapour pressure at the baking temperature; however, it is a slow process. Plasma cleaning procedures can rapidly remove contamination; however, they can damage some sensitive samples [24–25]. It should be noted that the growth rate of carbon layers under EB irradiation is also affected by the types of hydrocarbon molecules present in the vacuum chamber [26].

Normally, the deposition of carbon via focused EB irradiation is viewed as a simple addition of mass to the irradiated area. However, some studies suggest that this process may not always be so simple. Ueda and Yoshimura [27] reported the fabrication of free-standing nanowires on various metal surfaces (Al, Ag, Au, Cu, Pt, Ta, Ti, and W) via EBID with pump oil (hydrocarbons) as a precursor. It was observed that the substrate material greatly affects the growth rate of the nanowires, with the Cu surface providing the fastest growth. Energy-dispersive X-ray spectroscopy (EDX) revealed the presence of both carbon and substrate materials in the nanowires. The effect of substrate material on nanowire growth rate could be attributed to different adhesion coefficients between the surface and hydrocarbons. Results from EDX measurements strongly suggest that metal atoms or ions are capable of moving within carbon–metal nanowires under the influence of EB. High Ag ion mobility under electric fields or EB has also been reported in LiAlSi glasses [28] and TiO_2_ [29].

In one of our previous studies [30] we investigated the growth dynamics of nanodots on various metal surfaces (Al, Ag, Cu, Cr, and Mo) under focused EB irradiation in an SEM vacuum chamber. Similar to the previously discussed study, an influence of the surface material on the growth rate of the nanodots was observed. Metals with lower melting points (implying lower interatomic bond energy) produced higher nanodots at similar irradiation parameters. Conversely, metals with higher volume magnetic susceptibility produced much wider nanodots with a higher volume. A subsequent study [31] of surface irradiation on a shape memory alloy Ni40Ti60 suggests that surface expansion might take place during the growth of the nanodots. An additional deformation of the surface was observed around nanodots on an Ni40Ti60 surface, which is believed to be caused by the surrounding surface layer of twinned B19’ martensite being pulled up along the growth of the nanodot. After heating the Ni40Ti60 surface to 100 °C, where the shape memory effect is expected to be activated due to the phase transition from B19’ martensite to B2 austenite, the surface surrounding the nanodot was found to have been reverted to a flat surface. The surface expansion could be explained under the assumption that, under focused EB irradiation, carbon can also diffuse and accumulate within the metal layer, which would cause the surface metal layer to deform and expand.

There is a study about tuning micromechanical oscillators via EB irradiation which contradicts the model of EB-induced carbonization as a simple process of adding carbon mass to the irradiated area [32]. Doubly clamped beams made of Au85Pd15 were irradiated by EB and their resonance frequency increased with exposure time and beam current due to EB-induced carbonization. However, changing the position of the EB along the oscillator did not affect their resonance frequency, which should be the case if carbonization was happening locally.

There is a potential for carbon and carbon-metal based nanostructured systems to be used in different fields of nanotechnology [33–36]. Therefore, understanding the process of nanostructure formation from hydrocarbon deposition on metal surfaces via focused electron irradiation is important. In this work we sought to deepen that understanding by observing how changing individual irradiation parameters (beam current, focusing, angle of incidence, and amount of hydrocarbons) affects the growth of nanostructures on Ag surfaces undergoing irradiation by focused EB in point mode.

## Experimental

**Figure 1:**
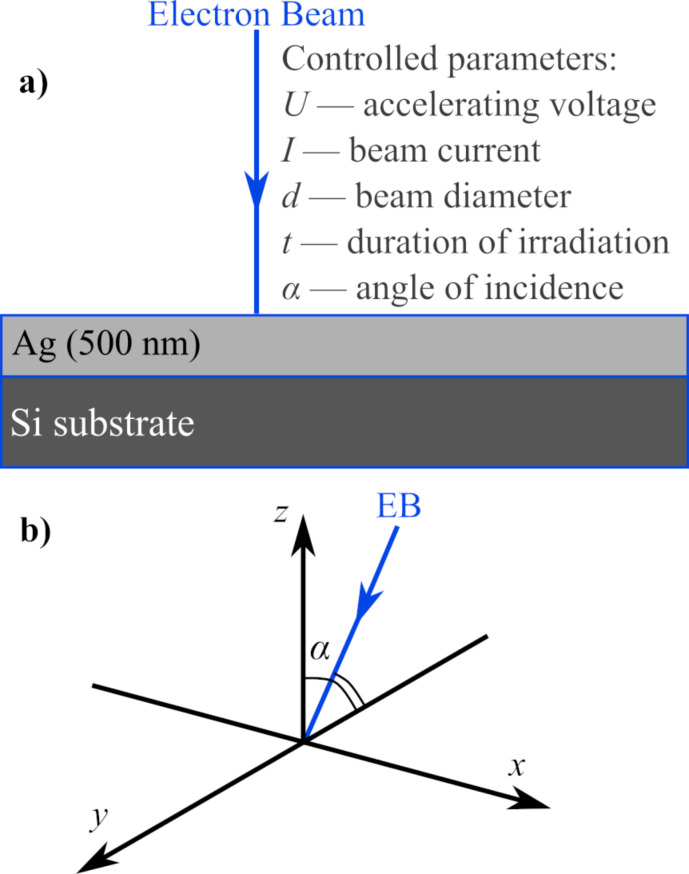
a) Structure of samples and experimental setup of their irradiation. b) Coordinate system for the angle of incidence of the electron beam; the surface of the samples is along the *xy* plane, and the angle of incidence is on the *yz* plane.

The samples were prepared by sputtering 500 nm thick Ag layers on Si(111) substrates via direct current (DC) magnetron sputtering. The samples were fixed to the SEM stub with colloidal Ag paint. The surface of the samples was irradiated with a focused EB with controlled parameters in point mode using a Tescan MAIA3 SEM (Figure 1a). Several samples were irradiated, each time one of the irradiation parameters was changed while the other parameters were kept constant. The accelerating voltage *U* was equal to 30 kV in all experiments and the angle of incidence α was equal to 0°, unless stated otherwise. Following irradiation, the height and volume of the structures formed in irradiated points were determined by non-contact atomic force microscopy (AFM) using the model Park NX10 AFM.

The first experiment was conducted with beam current *I* as the variable parameter ranging from 7 to 500 pA. However, changing the value of *I* also changed the beam diameter *d*, which is a function of *I* and the working distance (WD). The value of WD was adjusted to maintain *d* constant while changing *I*. Since the range of WD was limited by the movement of the SEM stage, it was impossible to fit the whole range of *I* in a single value of *d* without the risk of hitting the sample with the electron gun. Therefore, this measurement was split into two separate *d* values: 10 and 15 nm. In this experiment, the duration of the irradiation *t* was set to 40 s for each point.

The second experiment focused on testing the effects of moving the focus of the EB above or below the surface of the sample. This was done by focusing the EB on the surface of the sample and moving the SEM stage along the *z* axis for different irradiation points. The beam current *I*, the beam diameter *d*, and the duration of the irradiation *t* were respectively set to 40 pA, 10 nm, and 60 s for this experiment.

The third experiment explored how the angle of incidence α of EB could affect the formation of nanostructures on an Ag surface. The value of α was changed by tilting the SEM stage. The position of the EB relative to the sample surface is illustrated in Figure 1b. The EB was tilted along the *yz* plane over a 0–50° range and irradiation was carried out with different values of α along the *x* axis. The EB was refocused on the surface of the sample every time α was changed. For this experiment, *I*, *d*, and *t* were respectively set to 42 pA, 14 nm, and 60 s.

The fourth and last experiment considered the effects of hydrocarbon contamination in the vacuum chamber on the formation of nanostructures. This was done by irradiating the Ag surface with a focused EB before and after the nitrogen plasma cleaning procedure in the SEM vacuum chamber. The procedure lasted 5 min and the applied power was 20 W. The time was counted from the end of the cleaning procedure. The sample had to be removed from the vacuum chamber during the cleaning procedure since the N plasma cleaning procedure affected the Ag surface, most notably by forming a layer which produces thin film interference. The sample irradiation was repeated 15 min, 30 min, 1 h, and 2 h after the end of the cleaning procedure. For this experiment, *I* was 55 pA, *d* was 14 nm, and *t* was 30 s.

## Results

**Figure 2:**
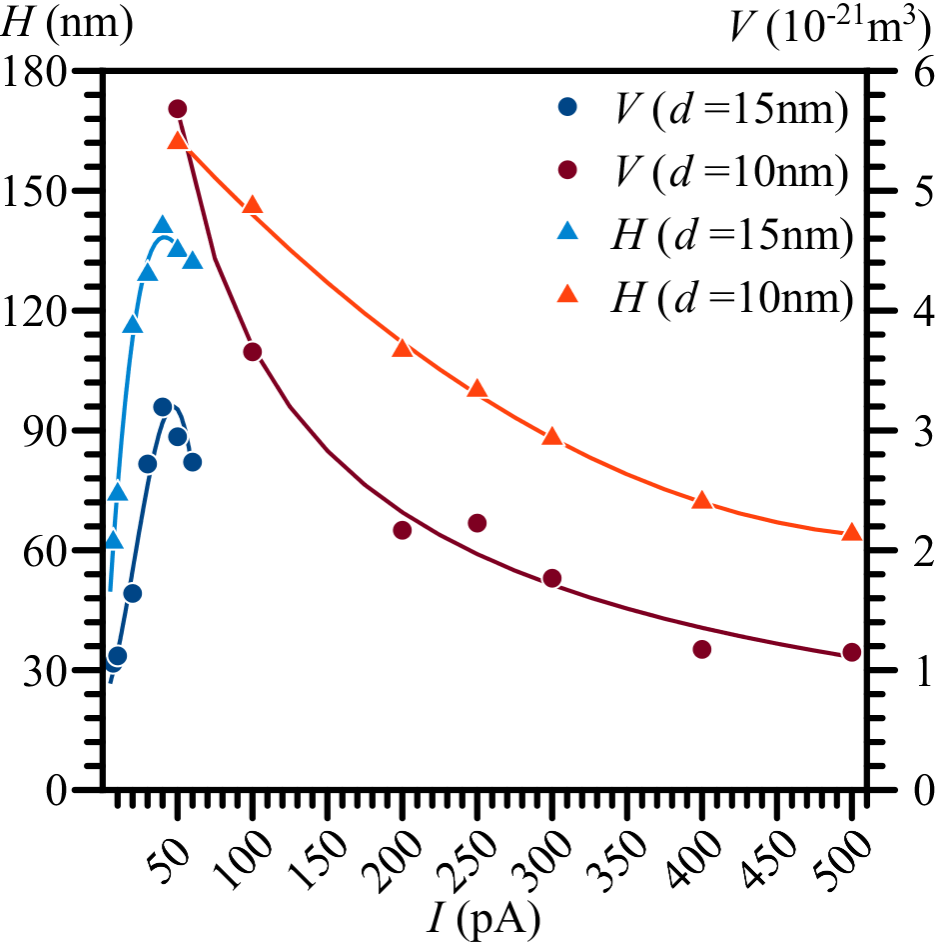
The volume and height of nanostructures on an Ag surface as functions of the electron beam current *I* and diameter *d*. Constant beam parameters are: *U* = 30 kV, *t* = 40 s, and α = 0°.

Figure 2 shows how the current and diameter of the EB affect the growth of nanostructures on an Ag surface. Over the EB current range of up to and around 40 pA, the height of the nanostructures sharply increased up to 140 nm with the increase of beam current when the beam diameter was 15 nm. When the EB current increased beyond 40 pA, the height of the nanostructures started to decrease at a slightly slower rate. Decreasing the EB diameter from 15 to 10 nm increased the height of the structures from 135 to 162 nm at an EB current of 50 pA. Increasing the EB current further, from 50 to 500 pA, resulted in a gradual decrease of the height of the structure from 162 to 64 nm when the beam diameter was 10 nm.

**Figure 3:**
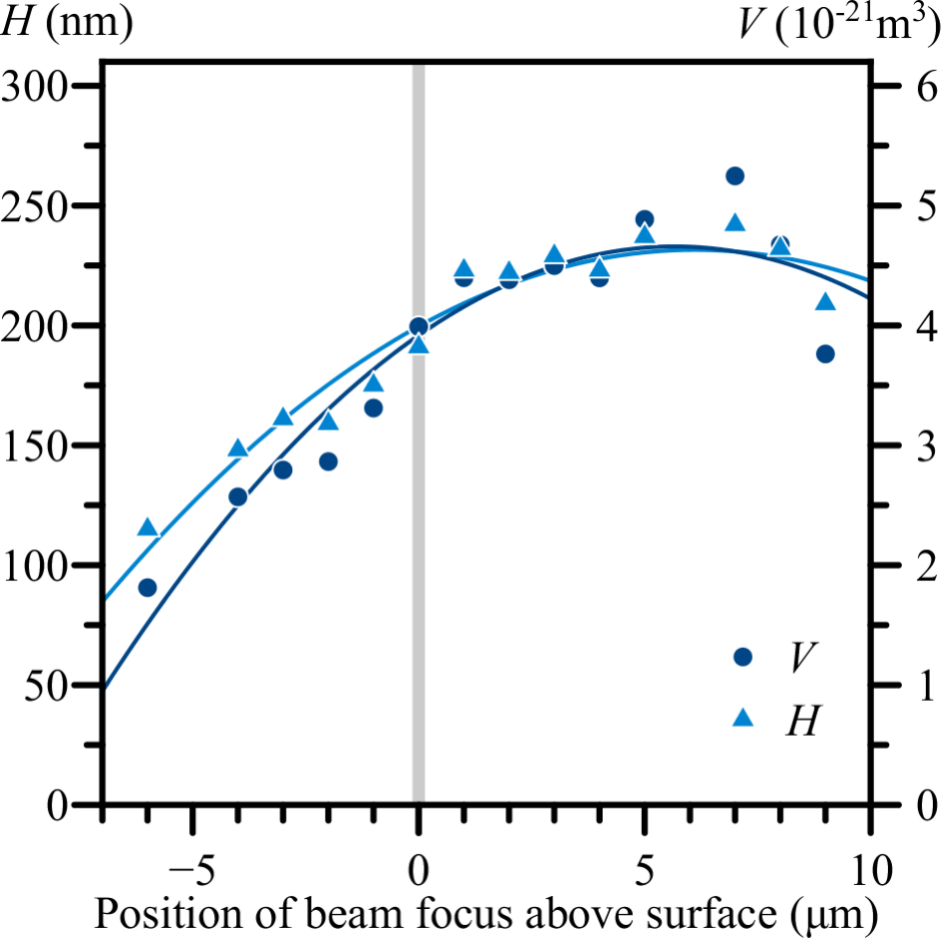
The volume and height of the nanostructures on an Ag surface as functions of the position of the focus of the electron beam relative to the surface. Constant EB parameters are: *U* = 30 kV, *I* = 40 pA, *d* = 10 nm, *t* = 60 s, and α = 0°.

Figure 3 shows the results of moving the EB focus above and below the surface of the sample. When the focus of the EB was placed under the surface of the sample, the height of the nanostructures gradually decreased from 200 to approx. 110 nm as the focus reached 6 μm below the surface. When the focus was placed above the surface, the height of the nanostructures increased up to approx. 230 nm as the focus reached 5–6 μm above the surface. When the focus of the EB was placed higher than 6 μm above the surface, the height of the nanostructures started to decrease again.

**Figure 4:**
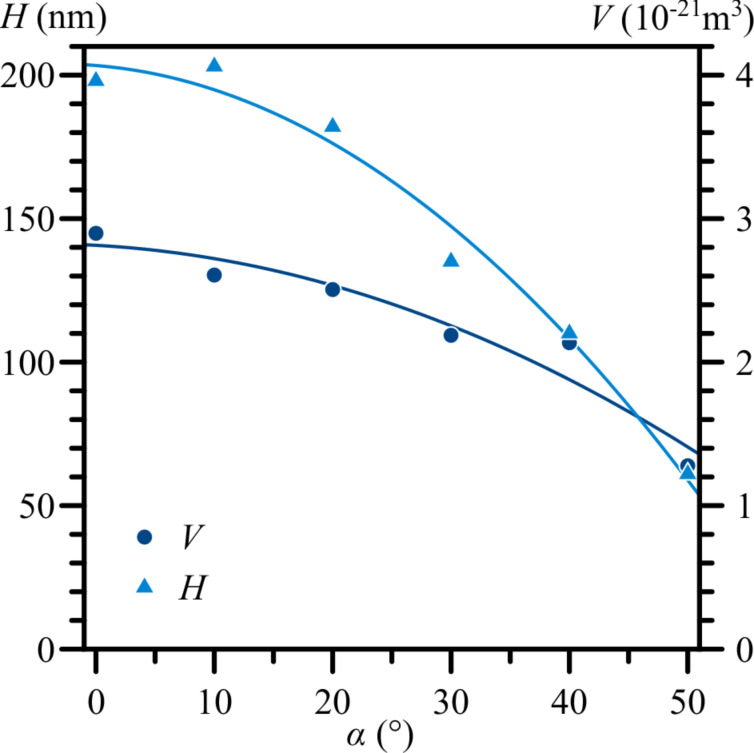
The volume and height of the nanostructures on an Ag surface as functions of the angle of incidence of the electron beam. Constant EB parameters are: *U* = 30 kV, *I* = 42 pA, *d* = 14 nm, and *t* = 60 s.

**Figure 5:**
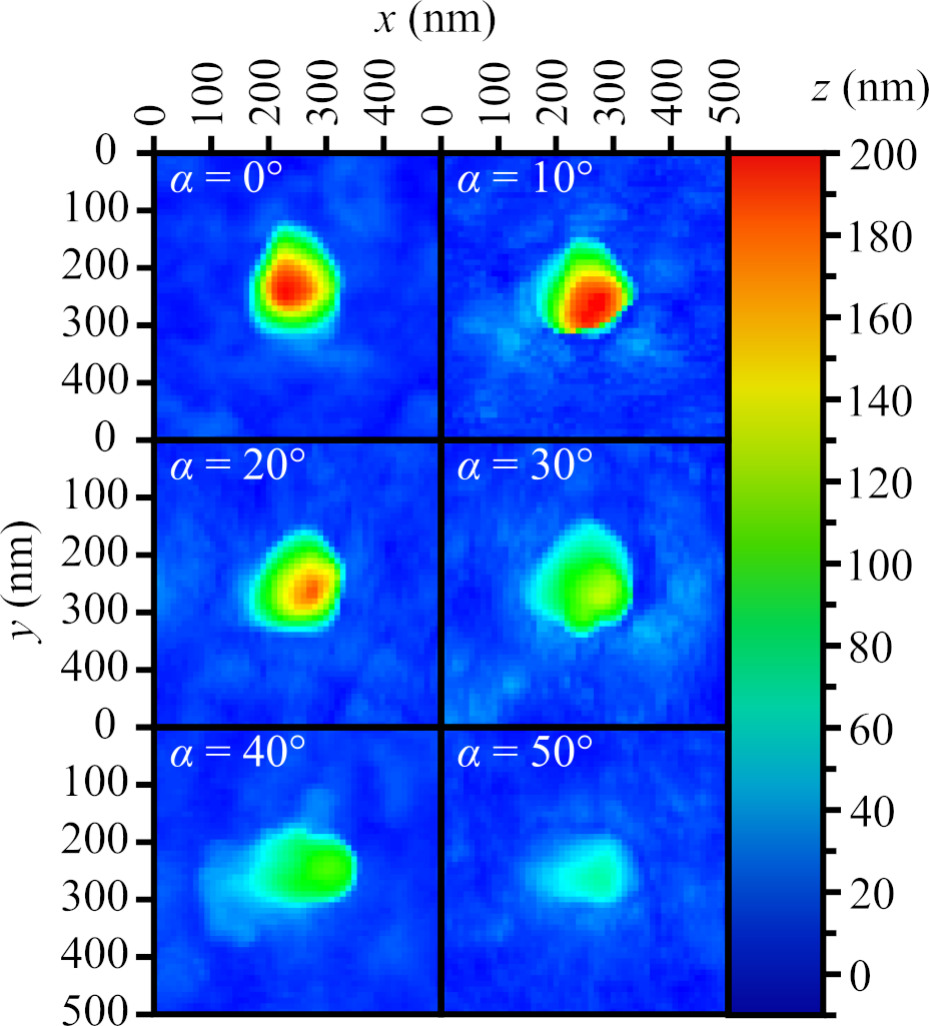
Atomic force microscopy images of the nanostructures on an Ag surface as a function of the angle of incidence of the electron beam. Constant EB parameters are: *U* = 30 kV, *I* = 42 pA, *d* = 14 nm, and *t* = 60 s.

The effects of changing the angle of incidence of the EB are presented in Figure 4 and Figure 5. The main effect observed was the height decrease of the nanostructure from 200 to 60 nm, as the angle of incidence increased up to 50° (Figure 4). We expected to observe a displacement of the peaks of the nanostructures towards the direction of the EB as the angle of incidence was increased; however, Figure 5 shows this did not happen. When comparing structures for α = 0° and 10°, the peak of the nanostructure with α = 10° was actually slightly displaced in the opposite direction.

**Figure 6:**
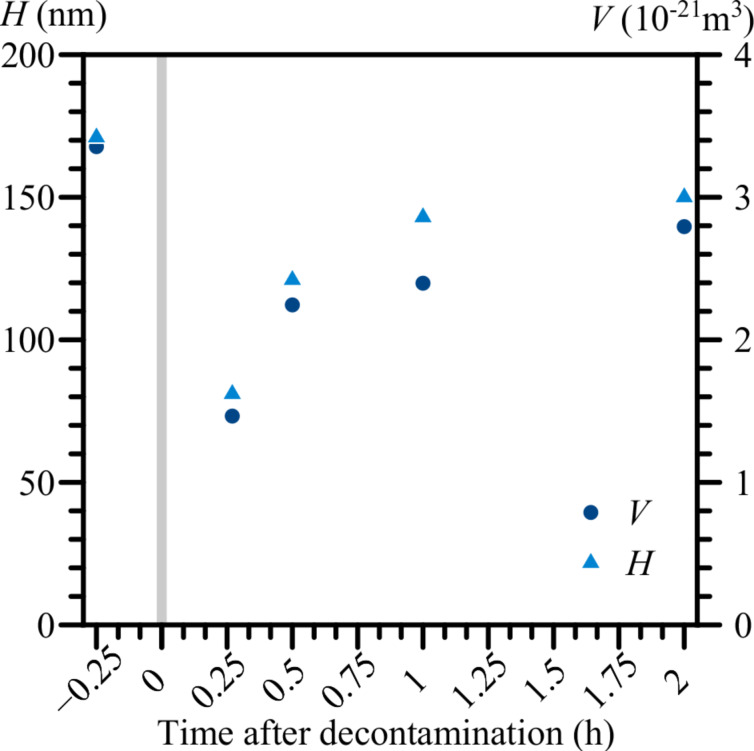
The volume and height of the nanostructures on an Ag surface as functions of time following chamber decontamination by nitrogen plasma cleaning. Constant EB parameters are: *U* = 30 kV, *I* = 55 pA, *d* = 14 nm, *t* = 30 s, and α = 0°.

**Figure 7:**
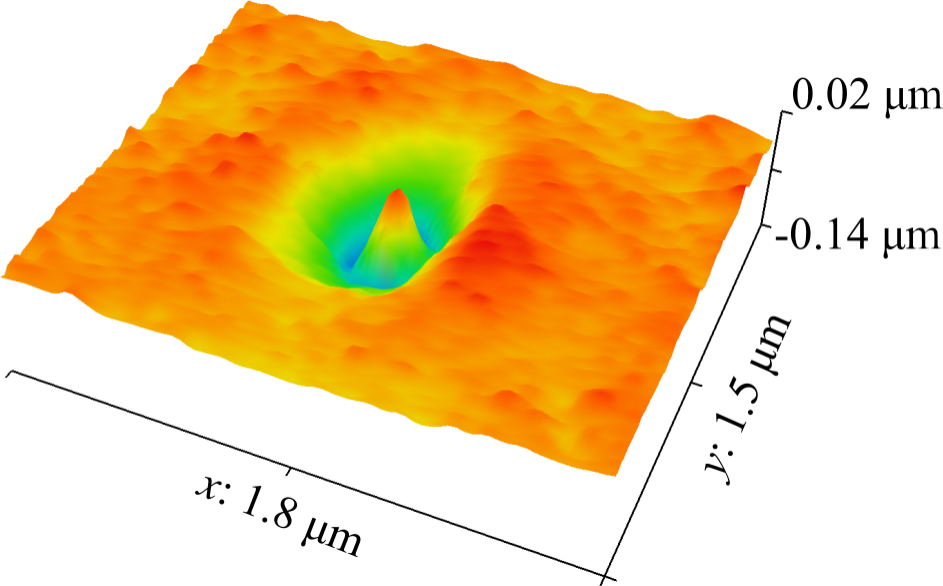
A representative AFM image of a nanostructure on an Ag surface after sustaining damage from N plasma cleaning. Constant EB parameters are: *U* = 30 kV, *I* = 52 pA, *d* = 11 nm, *t* = 60 s, and α = 0°.

The effects of vacuum chamber decontamination on nanostructure growth are presented in Figure 6. The nanostructures obtained before the cleaning procedure had a height of approx. 170 nm. The heights of the structures obtained 15 min after decontamination dropped to approx. 80 nm. Over time, the growth rate of the nanostructures gradually recovered, and 150 nm high structures were obtained 2 h after the cleaning procedure. It should be noted that the way plasma cleaning affected nanostructures from the initial experiment was quite interesting (Figure 7). Most of the material above the surface was removed and up to 140 nm deep moat-like depression was created around the point of EB impact.

## Discussion

Two noteworthy observations can be made according to Figure 2. The first would be the height increase of the nanostructure when the EB diameter was reduced from 15 to 10 nm, while maintaining the same beam current. This observation supports the theory described in our previous work [30] regarding the movement of positive metal ions within the electric field formed around a negatively charged EB, promoting nanostructure formation on metal surfaces. A smaller beam diameter would imply a higher current density and a stronger local electric field, resulting in a larger attractive force on the metal ions. The second observation is the existence of a curve peak around a beam current value of 40 pA. The shape of the curve under 40 pA can be rather easily explained by the beam energy and current density. A higher beam current generally means that the electric field around the beam focus is stronger and that the beam thus supplies more energy to the surface for its deformation and structure growth. However, why the curve peaked and started to decline at and above 40 pA is unclear. The most likely explanation would be the presence of another process which hinders the formation of nanostructures, such as, for example, the radiolysis of atoms due to strikes from high-energy electrons. It seems likely that interactions between the surface of the sample and high-energy electrons (radiolysis, ionisation, and breaking of atomic bonds) could promote the formation of nanostructures by making the surface more malleable. However, as the current density was increased, these interactions could start breaking down the formed nanostructures, limiting their resulting heights. The volume of the nanostructures appears to change proportionally to their height. It should be noted that the real values of height and volume of the nanostructures may slightly differ from the ones measured by AFM due to the tip convolution effect [37]; however, this should not affect the displayed trends.

The results presented in Figure 3 show that the height of the nanostructures on the Ag surface could be increased by elevating the EB focus by a few microns above the surface during the irradiation process. If the electric field, which causes the movement of metal ions, was centred around the focus of the EB where the negative charge density is the highest, moving the focus above the surface would have increased the electric field component in a positive direction along the *z* axis (Figure 1b) and resulted in an increased upward movement of ions. The opposite could likely be true for moving the EB focus below the surface of the sample, besides effectively increasing the beam diameter at the surface level. The decrease of the heights of the nanostructures when increasing the angle of incidence of the EB, as seen in Figure 4 and Figure 5, could be explained by a decreased component of electron energy/energy flow along the surface normal. The displacement of the nanostructure peaks away from the EB (Figure 5, α = 10°) could be related to the destructive effects of high-energy electrons at the EB point of impact.

Regarding the problem of hydrocarbon contamination in the vacuum chamber, Figure 6 clearly shows that the presence of hydrocarbons in a vacuum chamber enhances the growth rate of nanostructures during EB irradiation. Following the plasma cleaning procedure of the vacuum chamber, the growth rate of the nanostructures was cut by about a half and slowly recovered over time, as hydrocarbon concentrations returned to normal levels. The results from Figure 7 support the theory about EB-induced carbon diffusion within the metal substrate and may provide hints of the carbon distribution there. Under the assumption that carbon and carbon-containing silver areas are more susceptible to N plasma etching, we could theorize that carbon atoms in this particular case have reached up to 140 nm deep within the Ag layer. The carbon diffusion could have been caused by a number of reasons, radiolysis by collisions with electrons seeming like the most obvious one. It should be noted that N plasma treatment in this case also affected the clean Ag surface, turning it into an interfering thin film. We believe that controlled etching with plasma has the potential to explore the structure of metal–carbon systems. In this work, the hydrocarbon concentrations in the SEM vacuum chamber have been be considered as a constant, unless stated otherwise. Unfortunately, hydrocarbon contamination remains a difficult value to control and precisely quantify. This also means that nanostructures obtained in different devices with varying levels of carbon contamination may not exactly have the same size and purity, even if the EB parameters are set to be identical. On the other hand, carbon contamination in the produced nanostructures may not always be a disadvantage, particularly if the properties of the nanostructures could be controlled and modified in a beneficial way. This, however, would require further research on the various properties (electric, magnetic, optical, and mechanical) of nanostructures obtained through irradiation by a focused EB on metal surfaces.

## Conclusion

In this work we explored how varying different parameters of focused EB irradiation in point mode on Ag surfaces can affect the growth of nanostructures via the deposition of residual hydrocarbons. The optimal beam current for nanostructure growth was determined. Elevating the focus of the beam above the Ag surface increased the growth rate of the nanostructures, while lowering the focus below the Ag surface had the opposite effect. This observation can be used to support the theory that Ag ion movement occurs due to the electric field around the EB focus. Carbon diffused beneath the Ag surface after EB irradiation. We believe that a simplistic model of carbon mass deposition on a metal surface is not always sufficient to describe the growth of nanostructures on metal surfaces under focused EB. Diffusion of carbon and metal ions can occur under some conditions of irradiation, but further research is necessary to determine the concentration and distribution of the metal and carbon atoms within the obtained nanostructures.
